# Progress in the development of stabilization strategies for nanocrystal preparations

**DOI:** 10.1080/10717544.2020.1856224

**Published:** 2020-12-18

**Authors:** Jingru Li, Zengming Wang, Hui Zhang, Jing Gao, Aiping Zheng

**Affiliations:** Department of Pharmaceutics, Institute of Pharmacology and Toxicology of Academy of Military Medical Sciences, Beijing, China

**Keywords:** Nanocrystal, polymers, surfactants, solidification, stability, characterization

## Abstract

In recent years, nanocrystal technology has been extensively investigated. Due to the submicron particle size and unique physicochemical properties of nanocrystals, they overcome the problems of low drug solubility and poor bioavailability. Although the structures of nanocrystals are simple, the further development of these materials is hindered by their stability. Drug nanocrystals with particle sizes of 1∼1000 nm usually require the addition of stabilizers such as polymers or surfactants to enhance their stability. The stability of nanocrystal suspensions and the redispersibility of solid nanocrystal drugs are the key factors for the large-scale production of nanocrystal preparations. In this paper, the factors that affect the stability of drug nanocrystal preparations are discussed, and related methods for solving the stability problem are put forward.

## 1. Introduction

With the rapid development of combinatorial chemistry and high-throughput screening technologies, many potential drug candidates with satisfactory receptor targeting have emerged in recent years. However, due to the low water solubility of these candidate drugs, further preparation development is limited (Jermain et al., 2018). Drug nanocrystals are insoluble drug particles that form inhomogeneous water dispersions with particle sizes of 1∼1000 nm under the stability of surfactants or/and polymers. Different from other nano preparations such as liposomes, nanoparticles, and other solid lipid nanoparticles as the ‘carrier’ for drug delivery, drug nanocrystals have a simple composition, usually contain only pure drugs, do not require a carrier, and may include small amounts of stabilizers such as surfactants and a filling agent such as sucrose, thereby minimizing accessory-related toxicity (McKee et al., 2010; Barle et al., 2013); another advantage of the high drug loadings of drug nanocrystals is increased patient compliance. Therefore, drug nanocrystal technology has been widely investigated as a method for increasing the bioavailability of insoluble drugs.

Due to the unique advantages of nanocrystals, various pharmaceutical nanocrystals have been successfully commercialized (Möschwitzer, 2013). The production techniques are classified as either bottom-up (antisolvent precipitation) or top-down techniques (high-pressure homogenization, media milling, etc.) (Ahire et al., 2018). The bottom-up approach has not yet led to a product on the market; the marketed products are typically produced via a wet media milling or high-pressure homogenization technology. In 2000, Rapamune^®^ tablets of Sirolimus nanocrystals were marketed as immunosuppressants with 21% higher bioavailability compared to the oral solution (Zhou et al., 2016). An aprepitant nanocrystal, namely, Emend^®^, was introduced to the market in 2003 (Zhang et al., 2014; Roos et al., 2018), which showed increased absorption and reduced drug–food interactions compared with the micronized aprepitant, as well as improved bioavailability. Tricor^®^ (2004) and Triglide^®^ (2005) have significantly increased bioavailabilities compared to fenofibrate coarse and micronized suspensions with minimal impact on food intake (Sauron et al., 2006; Li et al., 2009). The emergence of nanotechnology has created a new prosperity in all fields, including chemistry, physics, and life sciences (Cai et al., 2020; Zhang et al., 2020), which provides a new direction for drug delivery system. In particular, nanotechnology drugs have great application prospects in tumor-targeted therapy (Chen et al., 2020; Pan et al., 2020; Zhai et al., 2020).

However, the instability of nanocrystals has been hindering their development and production. The instability of nanocrystal preparations is primarily due to the small particle size, and the high surface energy that is caused by small particles leads to thermodynamic instability, which eventually leads to aggregation and Ostwald ripening. In this paper, the influencing factors, characterization, and evaluation methods of the stability of nanocrystal preparations are reviewed, and the key and difficult points to be considered in the research and development process are discussed.

## 2. Causes of instability of nanocrystals

**Figure 1.**
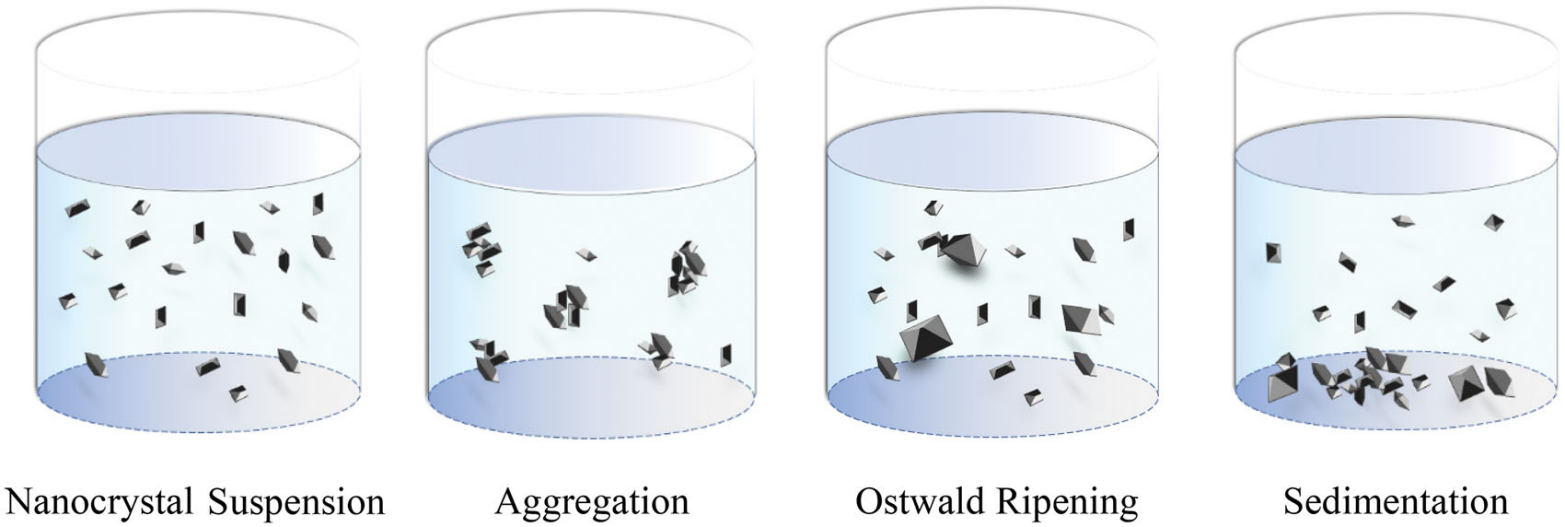
Instability mechanisms of nanocrystals.

Small particles have higher surface energy, so the particle size will increase to reduce the surface energy during storage. This section discusses the representative phenomena that affect the particle size of nanocrystals, including aggregation, sedimentation, Ostwald ripening, etc. (Figure 1).

### 2.1. Aggregation

A nanosuspension is a thermodynamically unstable heterogeneous water dispersion, and aggregation between crystals is one of the main reasons for its low stability. Particles in suspension exhibit Brownian motion, and they can collide, stick together, and coalesce due to the attraction between the particles and van der Waals forces (Berre et al., 1998). This phenomenon can be observed in the preparation and storage of nanocrystal suspensions. The aggregation of nanoparticles increases the particle size, broadens the particle size distribution, and, thus, reduces the solubility and dissolution rate of drugs.

### 2.2. Ostwald ripening

Ostwald ripening (crystal growth) is a phenomenon in which crystals of various particle sizes grow due to differences in solubility. According to the Ostwald–Freundlich equation, the preparation of an insoluble drug in a nanocrystal suspension could significantly improve the drug solubility. When the particle size is less than 1 μm, the drug solubility increases with the decrease of the particle size:

(1) $$\log\left(\frac{S_{2}}{S_{1}}\right)=2\sigma M(\frac{1}{r_{2}}-\frac{1}{r_{1}})/\rho RT$$

where S_1_ and S_2_ are drug solubilities with radii r_1_ and r_2_, respectively; σ is the surface tension between the solid drug and the liquid solvent; M is the relative molecular mass; ρ is the density of the solid drug; R is the molar gas constant; and T is the thermodynamic temperature.

Since small crystals have higher surface free energy, they have higher saturation solubility than large crystals, which leads to a drug concentration gradient between crystals. A smaller crystal interacts with a larger crystal, and the resulting diffused mass exchange causes the larger crystal to grow further and the smaller crystal to shrink and disappear (Singh et al., 2016).

### 2.3. Sedimentation

Sedimentation is a common cause of instability of nanosuspensions. In a suspension, particles of larger size settle naturally under the action of gravity, and their settling velocity follows Stokes’ law:

(2) $$v=2r^{2}（\rho_{1}-\rho_{2}）g/9\eta$$

where v is the settling velocity of a particle; r is the particle radius; ρ_1_ and ρ_2_ are the densities of the particle and medium; η is the viscosity of the dispersion medium; and g is the gravitational acceleration.

The sedimentation behavior of nanosuspensions can be divided into two types: flocculation and deflocculation. Flocculating suspensions are characterized by rapid and loose sedimentation, and sediments are easily redispersed. In contrast, deflocculation suspensions show a slow and dense settlement. Nanocrystal deposition is acceptable if the deposition rate is low and the sediments are easily redispersed. However, irreversible precipitation can lead to severe fluctuations in drug quality, thereby making it impossible for patients to obtain a uniform dose. Therefore, the inhibition of nanocrystal deposition is crucial for increasing the stability of nanocrystal drugs (Gao et al., 2011; Martínez et al., 2020).

## 3. Formability mechanism of nanocrystal suspensions

### 3.1. Drug-related factors

The formation of nanocrystal suspensions is influenced by the physical and chemical properties of the drugs, including polymorphism, log P, enthalpy, cohesive energy, etc. Not all drugs can form stable nanocrystal suspensions.

#### 3.1.1. Drug polymorphism

Many factors influence the molecular arrangement in drug nanocrystals, such as the solvent, temperature, and preparation process. The polymorphic forms and physical stability and solubility vary among arrangements (Shi et al., 2003). Therefore, in the formation of stable drug nanostructures, the polymorphic forms of drug nanocrystals must be considered. Compared with crystalline forms, amorphous forms are relatively unstable, and amorphous drugs are more soluble and prone to Ostwald ripening, thereby leading to an increase in the drug particle size (Lindfors et al., 2007).

#### 3.1.2. Drug hydrophobicity

The logarithm of the drug distribution coefficient, Log P, is the ratio of the concentration of an undissociated drug in the organic phase (usually *n*-octyl alcohol) to its equilibrium concentration in water. *N*-octyl alcohol is commonly used as an organic phase due to its similarity to the lipid layer of cell membranes. In contrast, water is used as an aqueous phase to simulate intracellular fluids. Log P is usually used to describe the hydrophilicity and hydrophobicity of a drug. When the concentration of the drug in the organic phase is 10 times the concentration in water, Log P is equal to 1. The larger the value of Log P, the higher the hydrophobicity.

**Figure 2.**
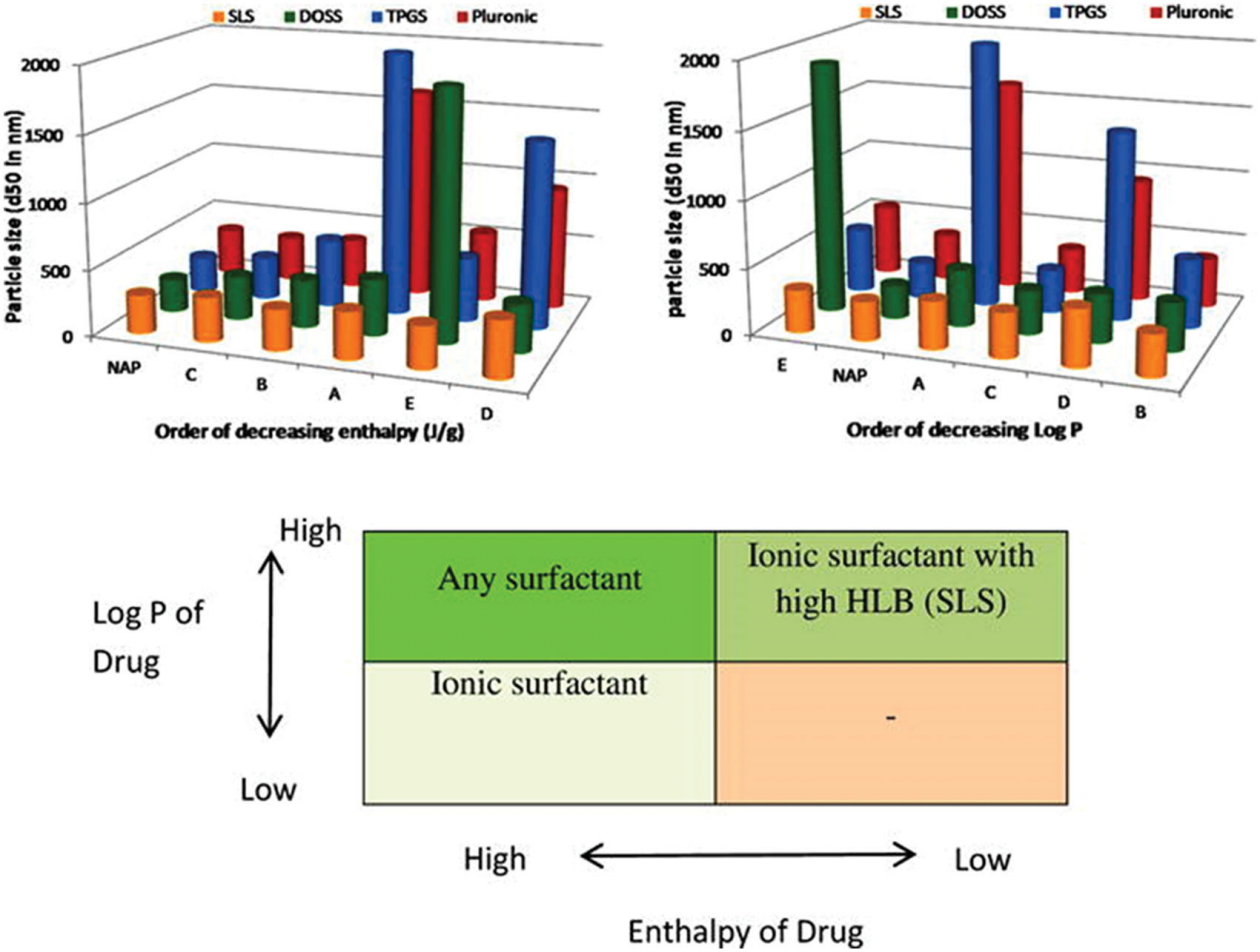
Proposed generic formulation of nanosuspensions based on drug properties. Reprinted with permission from George and Ghosh (2013). Copyright (2012) Elsevier B.V.

The main advantage of strong hydrophobic drugs over hydrophilic drug nanocrystals is that the stabilizers can cover the nanocrystals more easily. George & Ghosh (2013) found that drugs with high Log P values form highly stable nanosuspensions (Figure 2). The researchers believe that the attraction between the hydrophobic surface of the drug and the hydrophobic functional group of the stabilizer leads to the strong adsorption of the stabilizer on the drug surface and that hydrophobic drugs are more suitable than hydrophilic drugs for nanocrystal preparations because of the risk of reversible dissolution and precipitation.

#### 3.1.3. Drug enthalpy and cohesive energy

**Figure 3.**
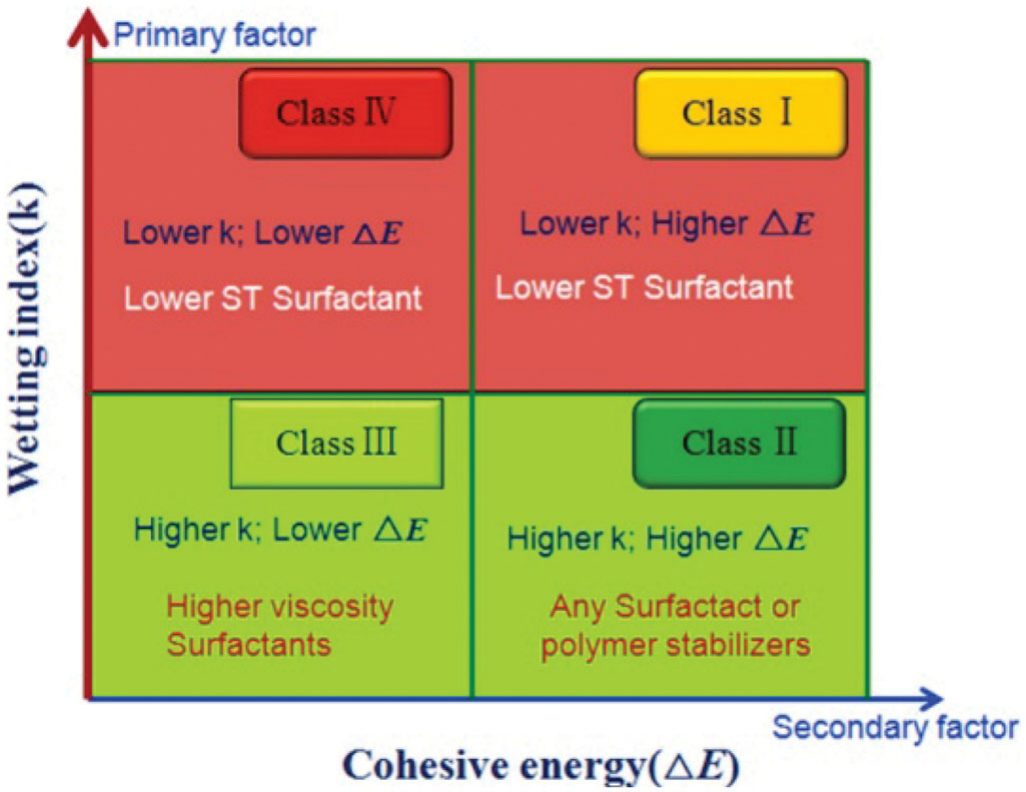
Proposed formulation design strategy of nanosuspensions based on drug and stabilizer properties. Reprinted with permission from Yue et al. (2013). Copyright (2013). Elsevier B.V.

Enthalpy represents the strength of the intermolecular interactions, and cohesion refers to the energy that is required by condensed matter to eliminate the intermolecular interactions. Both are important state parameters for characterizing the energy of a material system. George & Ghosh (2013) found that drugs with low enthalpy are prone to aggregation during the storage process. Due to the low enthalpy of these compounds, the crystal structures of drugs in water are easily destroyed, which may lead to a transition from a crystalline form to an amorphous form, thereby leading to the instability of the drug nanosystem. Yue et al. (2013) found that the surface hydrophobicity and cohesion of drugs are the main factors for the formation of nanocrystal suspensions (Figure 3). Under the premise that stabilizers and drugs can be wetting, drugs with high cohesion are more likely to form stable nanocrystal suspensions.

### 3.2. Stabilizing agent related factors

**Figure 4.**
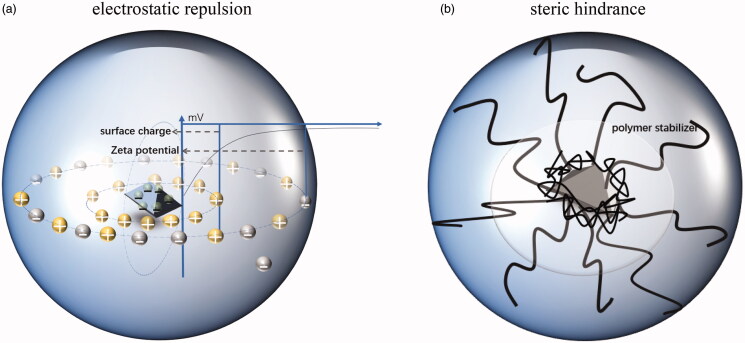
Action mechanisms of two types of stabilizers.

Stabilizers are essential for preventing nanocrystals from accumulating. The surface tension of drug nanocrystals is often very high, which leads to the facile aggregation of drug particles. The use of a suitable stabilizer can reduce the surface tension and prevent the aggregation of nanocrystals. As illustrated in Figure 4, ionic surfactants stabilize suspensions by initiating electrostatic repulsion between drug nanocrystals. In this case, when the stabilizer is adsorbed on the drug surface, a double electric layer is formed from the hydrophilic part of the stabilizer, and a charge is formed around the drug. When two drug particles are attracted to each other, they move closer to each other, and when the distance is reduced past a threshold, the two layers of the same charge repel each other and the particles separate, which eventually prevents polymerization. Polymers and nonionic surfactants maintain the stability of suspensions through spatial barriers, and they act as space stabilizers by adsorbing hydrophobic molecules on the surfaces of drug nanocrystals. The long hydrophilic chains of the polymers that are adsorbed on the nanocrystal surface extend further outward, thereby limiting the movement of drug particles to maintain the distance between drug particles.

While elucidating the stabilization mechanism of different stabilizers, their deficiencies are also exposed. The stability of nanosuspension system stabilized by electrostatic repulsion can be inhibited by the electrolytes or high acid conditions. Especially, oral drugs are exposed to an acidic gastric condition, the stable electrostatic interaction system may be destroyed due to the influence of electrolytes in gastrointestinal fluids (Rachmawati et al., 2016). The stability of the nanosuspension system stabilized by steric hindrance is not disturbed by charge ions, but the interaction between the stabilizer and the drug is more complex, the suitable polymer should be selected according to the physical and chemical properties of the drug (George & Ghosh, 2013). Suspensions containing high concentration polymers and drugs are often not conducive to the preparation of nanosuspensions because of their high viscosity (Medarević et al., 2018). It has been reported in many literatures that the stabilizers with different stabilization mechanisms have been applied to the preparation of nanosuspensions to produce a synergistic effect and obtain a stable nanosuspension system (Zuo et al., 2013; Toziopoulou et al., 2017; Medarević et al., 2018). In addition, there are also some uncommonly used stabilizers, such as whey protein isolate, soybean protein isolate, etc. (He et al., 2013), which have a strong affinity with drugs and stable adsorption on the surface of drugs, forming an effective space protective barrier. Some polyphenols, such as tannic acid and epigallocatechin gallate, have also been used in nano-drug delivery systems (Bartzoka et al., 2017; Luo et al., 2020; Su et al., 2020). Table 1 lists the common stabilizers classified based on the mechanism of stabilization. This section discusses the influence of the key properties of stabilizers on the development of stable nanosuspension formulations.

**Table 1.** Various types of stabilizers frequently used for stabilization of nanosuspensions.

Category		Stabilizers	Mechanisms
Surfactants	Ionic	SLS	Electrostatic repulsion
		Cetrimonium chloride	Electrostatic repulsion
		Dowfax 2A1	Electrostatic repulsion
	Nonionic	Poloxamer 188	Steric hindrance
		Poloxamer 407	Steric hindrance
		TPGS	Steric hindrance
		Tween 80	Steric hindrance
		Plantacare 2000	Steric hindrance
		Saponins	Steric hindrance
	Amphoteric	Lecithin	Steric hindrance
Polymers	Synthetic	PVP	Steric hindrance
		PVA	Steric hindrance
	Semisynthetic	HPMC	Steric hindrance
		HPC	Steric hindrance
		CMC-Na	Electrostatic repulsion
	Natural	Sodium alginate	Steric hindrance
		Chitosan	Electrostatic repulsion/ steric hindrance
Food proteins		Whey protein isolate	Steric hindrance
		Soybean protein isolate	Steric hindrance
		β-Lactoglobulin	Steric hindrance

#### 3.2.1. Molecular weight of the stabilizer

The hydrophobic end of the polymer stabilizer adsorbs on the surface of the drug nanocrystal, which can provide spatial stability, and stabilizers with higher molecular weight typically outperform stabilizers with lower molecular weight. The mutual attraction between drug nanocrystals that is caused by van der Waals forces leads to the aggregation of the nanocrystals. A long-chain polymer stabilizer can effectively induce spatial repulsion and prevent the aggregation of particles (Lee et al., 2005). A polymer stabilizer with a molecular weight of less than 5000 g/mol has difficult forming a spatial barrier for the mutual attraction between particles. In comparison, a polymer stabilizer with a molecular weight that exceeds 25,000 g/mol may lead to nanocrystal bridging due to the large molecular chain length (Lee et al., 2005; Choi et al., 2008; Peltonen & Hirvonen, 2010; Tuomela et al., 2016). The selection of a polymer of suitable molecular weight via experimental design is essential for the preparation of a stable nanosuspension.

#### 3.2.2. Hydrophilic and hydrophobic properties of the stabilizer

The hydrophilicity and hydrophobicity of a surfactant can be expressed by the hydrophilic lipid equilibrium (HLB) values (Pasquali et al., 2008; VermaGokhale et al., 2009). The HLB value of a hydrophobic surfactant is low while that of hydrophilic surfactant is high. To improve the stability of drug nanocrystals, the stabilizer should have sufficient affinity with the surfaces of the drug particles (Lee et al., 2005). When insoluble drugs show high hydrophobicity, the hydrophobicity of the stabilizer is the main driving force for the surface adsorption of the drug particles, which is crucial for the spatial stability and uniform dispersion of the drug particles (Van Eerdenbrugh et al., 2009). It is impossible to realize stability without adsorption, and it is also impossible to obtain a dispersed nanocrystal suspension. Moreover, the hydrophilicity of the stabilizer is important because most drug nanocrystals are dispersed in water and the hydrophilic portion of the stabilizer will be oriented toward water rather than the hydrophobic surface of the drug, thereby facilitating the inhibition of the drug nanocrystal aggregation. Hydrophilic molecules that contain electric charges can further stabilize drug nanocrystals through electrostatic repulsion between crystals, thereby providing sufficient space or charge stability for the drug nanocrystals. Ferrar et al. (2020) investigated the effects of 28 stabilizer formulations on the formability of drug nanocrystals using three insoluble drugs as models and found that the key factors that affected the stability of the nanocrystals were the amphiphilicity of the stabilizer and whether it had a sufficiently long hydrocarbon chain. Through a molecular model, it is shown that surfactant molecules with long and flexible hydrophobic chains can anchor on the surfaces of nanocrystals more effectively, thereby increasing the stability. Therefore, a stabilizer must have a suitable balance between hydrophilicity and hydrophobicity.

#### 3.2.3. Concentration of the stabilizer

It is necessary to prepare stable nanocrystals with a suitable stabilizer concentration. The optimal stabilizer concentration will maximize the adsorption affinity of the stabilizer to the drug surface (Deng et al., 2010). Spatial repulsion is induced by coating drug nanocrystals with stabilizers to prevent Ostwald ripening. Therefore, if the stabilizer concentration is insufficient, the drug particles cannot be effectively coated. If the drug particles are attached to the same stabilizer molecule, particle aggregation and bridging can occur, thereby resulting in reduced stability.

The stability of a nanosuspension is not directly proportional to the concentration of the stabilizer. Excessive stabilizer may lead to Ostwald ripening and decrease the stability over time. In addition, amphiphilic stabilizers in concentrations that exceed the critical micelle concentration (CMC) may lead to micelle formation. As the number of micelles increases, the micelles begin to compete for surface adsorption, and the total adsorption capacity at the drug interface begins to decrease, which will further undermine the stability of the nanosystem, thereby resulting in an increase in the particle size (Lo et al., 2009; Hui et al., 2011). Therefore, the use of a suitable stabilizer concentration is critical (Rangel-Yagui et al., 2005; Deng et al., 2010; Peltonen & Hirvonen, 2010; Hui et al., 2011).

### 3.3. Combined action factor

#### 3.3.1. Drug solubility in a stabilizer solution

The solubility of a drug is affected by the type of stabilizer that is used. When the solution of stabilizers increases the solubility of drug nanocrystals, the stability of these crystals decreases over time, thereby leading to the growth of the nanocrystals. For example, a study showed that PVP K30, Pluronic F68, and HPMC had no significant effect on ibuprofen solubility (VermaGokhale et al., 2009), and stable nanocrystal suspensions were obtained; however, as stabilizers, SLS, Twine 80, and Pluronic F127 increased the solubility of ibuprofen, thereby resulting in instability of the nanosuspensions and increased particle size during storage. Ghosh et al. (2011) reported similar results in a study on the use of the wet grinding process to improve the bioavailability of insoluble drugs. As 1% SLS increased the solubility of drugs, it also exacerbated the Ostwald ripening phenomenon. Therefore, the stabilizers with the weakest influence on the drug solubility are the first choices for the preparation of a nanosuspension.

#### 3.3.2. Surface energies and specific interactions of the drug and stabilizer

The interactions between drug nanocrystals and polymer stabilizers depend mainly on their respective surface energies. Especially when drug nanocrystals are dispersed in water, they have large surface area and high surface tension due to their small particle size and strong hydrophobicity. Therefore, drug nanocrystals exhibit higher surface free energy, and their dispersion becomes unstable, thereby leading to aggregation, solidification, or crystal growth (Verma et al., 2011).

To reduce the surface energy of drug nanocrystals and improve the stability of drug nanocrystals, it is necessary to humidify or hydrate the surfaces of the drug nanocrystals. The surface of a drug nanocrystal can be hydrated and modified by various materials to reduce the surface free energy (Gong et al., 2017; Wang & Gong, 2017a, 2017b). Hydrophilic polymers are commonly used to hydrate nanocrystal surfaces because they can interact strongly with surrounding water molecules (Choi et al., 2005).

In a study that analyzed the effects of polymer stabilizers on the stability of drug nanocrystals, seven drugs were wet-comminuted to form nanocrystals (Choi et al., 2005), and hydroxypropyl cellulose (HPC) and polyvinylpyrrolidone (PVP) were used as stabilizers. The results demonstrate that a drug with a surface energy that is similar to that of PVP can form stable nanocrystals effectively. Due to the strong interactions between drug nanocrystals and stabilizers, the use of polymer stabilizers that are similar in surface energy to the drug usually results in drug nanocrystals of stable and uniform particle size (Lee et al., 2008). The surface energies of drugs and stabilizers can be assessed using ‘static contact angle measurements’ (Choi et al., 2005; Lee et al., 2008) (see the subsection on the contact angle measurement below for details).

#### 3.3.3. Effects of dispersion media

To form a stable nanosystem, the temperature and viscosity of the dispersion medium must be suitable. The Stokes–Einstein equation can be used to explain the influence of the temperature and viscosity on the stability of the nanosuspension:

(3) D=kT/(6ηπr)

where D is the diffusion coefficient, k is the Boltzmann constant, T is the thermodynamic temperature, η is the viscosity, and r is the radius of the spherical particle (Zwanzig & Harrison, 1985; Harris, 2009).

According to the equation, the stability of the nanosystem is negatively correlated with the temperature and positively correlated with the viscosity of the medium. According to the Stokes–Einstein equation, high viscosity reduces the diffusion velocity of drug particles and, thus, stabilizes the nanosuspension (Milewski et al., 2010). The formation of the hydrophobic interaction between the nanocrystal system and the stabilizer is a negative entropy process. The higher the temperature of the nanocrystal system, the lower the stability of the system and the more likely the nanocrystal drugs are to aggregate. However, an increase in the temperature will lead to a decrease in the viscosity and an increase in the diffusion coefficient, which is very unfavorable for the interactions between particles in the nanosystem (Kakran et al., 2012). However, in a study that compared surfactants with polymer stabilizers, it was found that although surfactants have low viscosity and high surface activity, their stability is higher (Van Eerdenbrugh et al., 2008). The polymer stabilizer with a high viscosity has a poor effect on the preparation of stable nanocrystals, for which the main reason is that the high viscosity inhibits the decrease of the particle size in the preparation process of the nanocrystals.

### 3.4. Characterization and evaluation of the nanosuspension

#### 3.4.1. Contact angle measurements

**Figure 5.**
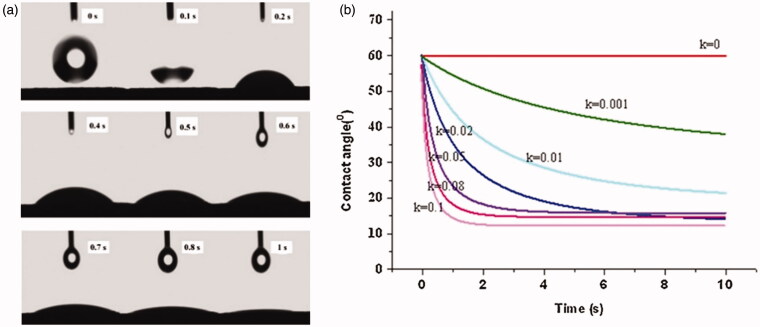
(a) The measurement process of the tangent of a droplet on a disk surface. (b) A schematic diagram of the wetting characterization of models with various k values. Reprinted with permission from Yue et al. (2013). Copyright (2013) Elsevier B.V.

Contact angle measurement is a method for measuring the wettability of a stabilizer. The smaller the contact angle, the higher the wettability. The contact angle of a stabilizer solution can be measured by compressing a small amount of powder to form a disk. Yue et al. (2013) evaluated the wettability of drugs through contact angles. Drugs with small contact angles and satisfactory wettability easily form stable nanosuspensions (Figure 5). Pardeike & Müller (2010) used the contact angle as the standard for the formula selection of a nanosuspension stabilizer. Purified water showed a contact angle of 51.6° on the compressed PX-18 disk. With 0.1% (w/v) Tween 80 solution, the contact angle was reduced to 23.2° (Table 2). Therefore, Tween 80 was selected as the stabilizing agent for PX-18 nanosuspensions. In another study, in which various stabilizers were screened for the preparation of miconazole nanosuspensions, the contact angles between the stabilizer solutions and the drug were determined (Cerdeira et al., 2010). The contact angle between miconazole and pure water exceeded 140°. The contact angle was determined to be 43° for a 2.5% HPC-LF and 0.1% SLS solution. However, miconazole had a large contact angle with PVP/SDS and Poloxamer solutions, which indicated poor wettability of the drug. The nanocrystal size was smaller when the stabilizer system with the lowest contact angle was used, which further demonstrated the practicability of the method.

**Table 2.** Contact angles that were obtained for purified water and 0.1% (w/v) surfactant/stabilizer solutions on compressed disks of PX-18 $(n=3,\;\overset{-}{x}\pm \text{SD}).$

Liquid	Contact angle (°)
Purified water	51.6 ± 0.6
Brij 56	30.5 ± 1.3
Inutec SP1	32.8 ± 0.6
Lipoid E80	38 ± 0
L.A.S.	26 ± 1
Nontanov 202	35 ± 0.6
Phospholipon 80	37.8 ± 0.8
Plantacare^®^ 2000	25.6 ± 0.6
Pluronic F68	28 ± 0
Tagat S	29 ± 0.6
TegoAcid S40P	42.3 ± 0.6
Tween 80	23.2 ± 0.3

Reprinted with permission from Pardeike & Müller (2010). Copyright (2010) Elsevier B.V.

#### 3.4.2. Micromorphological characterization

**Figure 6.**
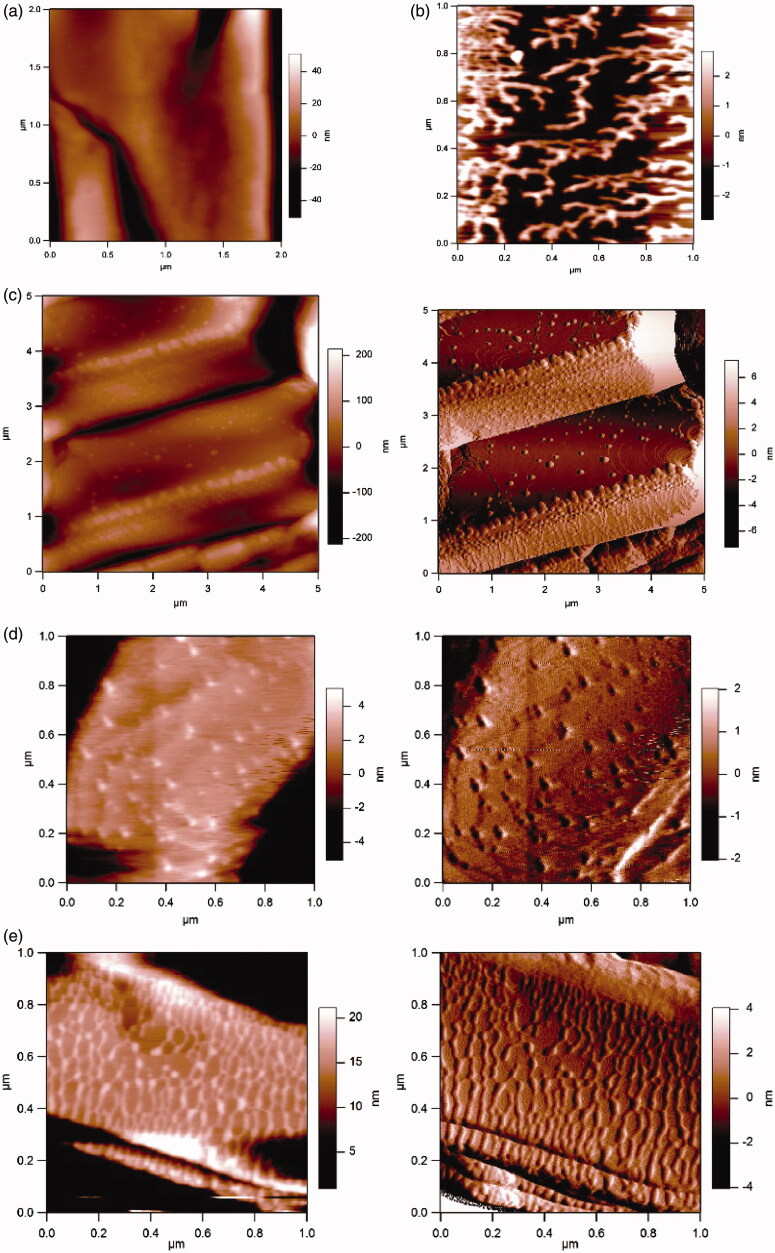
AFM images of various polymers that are adsorbed on an ibuprofen surface. Reprinted with permission from Verma et al. (2009). Copyright (2009) American Chemical Society. (a) Height image of bare ibuprofen surface captured in air using intermittent-contact mode. (b) Height image of HPMC adsorbed on ibuprofen surface captured in air using intermittent-contact mode. (c) Height (left) and amplitude (right) AFM images of PVP adsorbed on ibuprofen surface captured in air using intermittent-contact mode. (d) Height (left) and amplitude (right) AFM images of Poloxamer 188 adsorbed on ibuprofen surface captured in air using intermittent-contact mode. (e) Height (left) and amplitude (right) AFM images of HPC adsorbed on ibuprofen surface captured in air using intermittent-contact mode.

Atomic force microscopy (AFM) is an important visualization tool for nanocrystals. It enables the qualitative and quantitative analysis of the physical properties of nanocrystals, such as the size, surface structure, roughness, and morphology. The interaction forces between atoms and molecules are used to observe the surface morphology of an object and provide a three-dimensional surface map. Compared with scanning electron microscopy (SEM) and transmission electron microscopy (TEM), it has many advantages: Electron microscopes can only provide two-dimensional images, while AFM can capture three-dimensional images of nanocrystal surfaces without any special processing of the sample. Atomic force microscopy has proved to be a valuable tool for visualizing and quantifying pharmaceutical nanocrystals in preparations. In addition to precise size measurements, AFM can easily provide information about the shape and structure of nanoparticles that cannot be obtained by light scattering or other methods (Shi et al., 2003; Du et al., 2015). In addition, the method can be used to evaluate the interactions between the stabilizer and the surfaces of the drug particles, and the resulting affinity can be a satisfactory indicator of the stability of the nanocrystal preparation with the stabilizer. Verma et al. (2009) used AFM technology to screen the stabilizers in ibuprofen nanocrystal formulations (Figure 6). The captured AFM image clearly shows that on the ibuprofen particle surface, the polymerization chains of HPMC and HPC are fully unfolded and adsorbed on the ibuprofen particle surface. The strong interactions between HPMC/HPC and ibuprofen drug particles strongly suggest that both polymers are suitable for the formation of stable ibuprofen nanosuspensions. In contrast, the AFM images of PVP and Poloxamer show incomplete surface adsorption of ibuprofen particles, which results in low stability of the nanocrystal preparations that are obtained using PVP and Poloxamer.

#### 3.4.3. Particle size distributions of suspensions

The polydispersity index (PDI) represents the change of the particle size distribution of a nanocrystal suspension and is affected by its physical stability. Under normal circumstances, a PDI value of 0.1 ∼ 0.25 corresponds to a narrow particle size distribution, which indicates a stable nanocrystal suspension system, while a PDI value of >0.5 correspond to a wide particle size distribution range (Shah et al., 2017). Ensuring a narrow particle size distribution is an effective method for reducing the concentration gradient and the differences in the saturation solubility of drug nanocrystals. When drug nanocrystals have a wide particle size distribution, Ostwald ripening is more likely, which leads to decreases in the drug solubility and the dissolution rate and, ultimately, to a decrease in the bioavailability. Therefore, maintaining a narrow particle size distribution of drug nanocrystals is highly important for ensuring the stability of a drug nanocrystal suspension.

Photonic correlation spectroscopy (PCS) is one of the most commonly used particle size characterization techniques. It uses the principle of dynamic light scattering to evaluate the average particle sizes of nanocrystals in terms of Z-value, particle size distribution, and zeta potential (which refers to the potential of the shear plane). The PDI values range from 0 (monodispersed particles) to 0.500 (polydispersed particles) and are used to monitor the physical stability of nanocrystals. PCS has a narrow range of measurements (e.g. from 3 nm to 3 μm) and is not suitable for large particle size measurements. When the particles are large, they are measured via laser diffraction (LD), which measures a large range of particles (0.02–2000 µm) that depends on the type of instrument that is used. The data that are measured via PCS and LD are not similar in terms of granularity because the LD data are based on the volume distribution, whereas the PCS data are the weighted light intensity values. LD only measures the particle size distribution, whereas PCS also measures the average particle size and zeta potential, which can be used to convert strength data into volume and quantity distributions. If nanosuspensions are used intravenously, it is necessary to use the Coulter counting method. Since the smallest capillaries are 5 µm in size, there is a risk of capillary blockage if any particles that are larger than 5 µm are present in the intravenous formulation. Coulter’s counting method provides the absolute number of particles per unit volume at various size levels; hence, the number of nanoscale particles is strictly controlled.

Keck (2010) found that the dissolution of nanocrystals during measurement significantly affected the size results that were obtained. When an unsaturated medium or microparticle saturated medium is used, the sample will dissolve, the dissolution will be unstable, and the results will be unreproducible. If the particle sizes of nanocrystals are to be analyzed, the dispersion media should be pre-saturated with the nanocrystals because the solubility of the nanocrystals exceeds that of micro-sized drugs. In the early stage of formulation development, it should be confirmed whether the particle size analysis method requires a pre-saturated dispersion medium. The characterization of nanoparticles using both dynamic and static light scattering techniques can yield meaningful results if the necessary prerequisites are satisfied. Via the development and validation of a reasonable particle size detection methodology, misleading studies can be avoided, and the stability and instability of nanocrystals can be reliably distinguished at an early stage of development.

#### 3.4.4. Zeta potential in suspension

The zeta potential (ζ) is the main factor that affects the physical stability of nanocrystal suspensions. It is a measure of the charge on the shear surfaces of particles and reflects the physical stability of colloidal systems. When the absolute zeta potential of the drug nanocrystals is very small, the gravitational attraction between the particles exceeds the electrostatic repulsion, thereby causing nanocrystal aggregation. Typically, a zeta potential of 30 mv is required for obtaining an electrostatically stable nanocrystal suspension. The zeta potential of a suspension can be used to predict the storage stability, and particles with sufficient zeta potentials are difficult to aggregate due to electrostatic or spatial repulsion between the particles.

The zeta potential represents the stability of a nanosuspension; hence, it is necessary to evaluate the level of the zeta potential value reasonably. When a polymer is used as a stabilizer, the zeta potential on the nanocrystal surface depends more strongly on the polymer concentration than on the surfactant concentration; thus, the absolute potential value must be no less than 20 mV. In a study, the zeta potential of a glyburide nanosuspension that was stabilized by HPMC and SLS depended more strongly on the polymer concentration than on the surfactant concentration (Singh et al., 2011). HPMC is a nonionic polymer, and SLS is an anionic surfactant. When the polymer concentration is low, the particle surface of the drug is not highly densely covered by the polymer; as a result, the anionic surfactant can more easily reach the surface of the drug and the nanocrystal surface, and the zeta potential increases with the increase of the concentration of SLS. However, at a higher percentage of HPMC, the nanocrystal surface potential is not significantly affected by the concentration of SLS. Similar results were obtained in another study in which the zeta potential of a meloxicam suspension depended more strongly on the polymer concentration than on the surfactant concentration (Singare et al., 2010). Nanosuspensions typically realize stability through the synergistic action of polymer stabilizers and charge stabilizers. Therefore, for the polymers and charge protectors that are used to prepare nanocrystal suspensions, the optimal balance between the electrostatic repulsion of the zeta potential and the spatial stability that is provided by the polymer should be realized.

#### 3.4.5. Storage stability

The stability of a nanosuspension can be evaluated experimentally under various storage conditions. The stability of the nanocrystals will be assessed according to their size, polydispersity index (PDI), and zeta potential (Geng et al., 2017; Gol et al., 2018). In one study, miconazole nitrate nanocrystal suspensions were stored at refrigerated (4 °C), room (25 °C) and hyperthermal (40 °C) temperatures for further investigations (Pyo et al., 2017). The particle size and PDI of the nanosuspensions that were stabilized by Tween 80 did not change when stored at 4 °C and showed almost no change at 25 °C. However, the particle size and PDI both increased during storage at 40 °C. Via optical microscopy, the presence of needle-shaped crystals was observed, and the Feret diameter of approximately 5 μm was outside the measurement range of PCS and, thus. could not be detected. When Poloxamer 407 was used as a stabilizer, the particle size and PDI did not increase at 4 °C or 25 °C over 3 months, while particle growth was observed at 40 °C, but the increase was significantly less than that of the Tween 80 stable suspension.

## 4. Solidification of nanocrystal suspensions

Solidification is one of the stabilization strategies, and solid preparations are more stable than liquid preparations. The solidification of nanocrystal suspensions can reduce the generation of unstable factors of nanocrystals such as aggregation and Ostwald ripening; hence, prepared nanocrystal suspensions are usually converted into the solid state. Then, the solid powders are converted into other dosage forms, such as sterile powder for injection, oral tablets, and capsules (Wang et al., 2013).

### 4.1. Solid method of nanocrystal suspension

The solidification process is a key step in the formation of the final product. The solidification methods include spray drying, freeze drying, electrostatic spray drying, and the use of an aerosol flow reactor, among others (Chan & Kwok, 2011; Ho & Lee, 2012). In addition, a type of fluidized bed coating technology has been applied in the industry. Fluidized bed coating of pellets is a one-step pelletizing method in which a nanocrystal suspension is dried and wrapped around the cores of pills. The pellets can be used to realize satisfactory fluidity, which is conducive to tablet compression and capsule filling.

Spray drying and freeze drying are two main curing methods. To reduce the time and energy consumptions, spray drying is more widely used in the pharmaceutical industry than freeze drying. However, spray drying is not suitable for heat-unstable drugs, and freeze drying is the preferred technique for such drugs. The aggregation of nanoparticles should be minimized during solidification. In a nanocrystal suspension, stabilizers provide ionic or spatial stability by adsorbing onto the surfaces of the drug nanoparticles, thereby preventing nanoparticle aggregation. The solidification of nanocrystal suspensions may result in drying and solidification of the stabilizers, which may lead to unstable and irreversible aggregation of the drug nanoparticles (Chaubal & Popescu, 2008). Medarević et al. (2018) found that the spray-dried solidified carvedilol nanocrystals exhibited satisfactory redispersability when in contact with water, while strong agglomeration during freeze drying prevented the redispersion of carvedilol nanocrystals after freeze drying. Therefore, a reasonable solidification method should be selected (Niwa et al., 2011; Wang & Gong, 2017a). The dissolution rates of dry powder in water differ among curing methods. Salazar studied the effects of spray drying, freeze drying and wet granulation on the dissolution rates of glibenclamide nanoparticles (Salazar et al., 2013). The results demonstrated that the dissolution rates were highest for spray drying, moderate for freeze drying, and lowest for wet granulation. Table 3 presents case studies on the solidification of nanocrystal suspensions.

**Table 3.** Case studies of preparation and solidification processes of nano suspensions.

Number	Drug	Nanosuspension			Solidification		Reference
		Method	Polymer	Surfactant	Method	Dispersants/protectants	
1	Nifedipine	Wet media milling	HPC-SSL	Poloxamer 407	/	/	(Patel et al., 2018)
2	Fenofibrate	Wet media milling	HPMC	SLS	/	/	(Knieke et al., 2013)
3	Fenofibrate	Wet media milling	HPMC-E5	SLS	Spray drying	Lactose; sucrose; maltose; glucose; mannitol	(Zuo et al., 2013)
4	Fenofibrate	Wet media milling	HPMC	SLS	Fluidized bed coating	d-Mannitol	(Knieke et al., 2015)
5	Fenofibrate	Antisolvent precipitation	Tragacanth	/	/	/	(Zhang, H. et al., 2014)
6	Itraconazole	Wet media milling	HPMC E5	SLS	Fluid bed coating	HPMC VLV; copovidone	(Parmentier et al., 2017)
7	Myricetin	High-pressure homogenization	HPMC; HP-b-CD	TPGS; soya lecithin; Poloxamer 188	/	/	(Hong et al., 2014)
8	Naproxen	Wet media milling	HPMC E15	Dowfax 2A1	Spray drying	/	(Kumar et al., 2014)
9	Naproxen	Wet media milling	HPMC E15	Tween 80	/	/	(Kumar & Burgess, 2014)
10	Indomethacin	Wet media milling	HPMC E15	Dowfax 2A1	Spray drying	/	(Kumar et al., 2014)
11	Indomethacin	Wet media milling	PEG	Poloxamer188; Poloxamer 407; ween 80	/	/	(Liu et al., 2011)
12	Indomethacin	Wet media milling	/	Dowfax 2A1	Spray drying; Freeze drying	Sucrose; lactose; maltose; trehalose; mannitol; Ficoll PM70; maltodextrin	(Kumar et al., 2014)
13	Griseofulvin	Wet media milling	HPC-SL	SLS	/	/	(Afolabi et al., 2014)
14	Griseofulvin	Wet media milling	HPC-SL	SLS	Fluid bed coating	Mannitol	(Bhakay et al., 2013)
15	Griseofulvin	Wet media milling	HPC-SL	SLS	Fluidized bed drying; Spray drying	Mannitol	(Bhakay et al., 2014)
16	Azodicarbonamide	Wet media milling	HPC-SL	SLS	Fluidized bed drying; Spray drying	Mannitol	(Bhakay et al., 2014)
17	Griseofulvin	Wet media milling	HPC-SL	SLS	/	/	(Bilgili & Afolabi, 2012)
18	Phenylbutazone	Wet media milling	HPC-SL	SLS	Fluid bed coating	Mannitol	(Bhakay et al., 2013)
19	Glimepiride	Wet media milling	HPC-SL; HPMC	Poloxamer 188	Spray drying	Mannitol	(Medarević et al., 2020)
20	Glibenclamide	Wet media milling; high-pressure homogenization	/	Docusate sodium salt (DSS)	/	/	(Salazar et al., 2012)
21	Glyburide	Wet media milling	HPMC 6 cps	SLS	Spray drying	/	(Singh et al., 2011)
22	Miconazole	Wet media milling	HPC-LF	SLS	/	/	(Cerdeira et al., 2010)
23	NVS-102	Wet media milling	HPMC 3 cps	TPGS	/	/	(Ghosh et al., 2011, 2012)
24	PX-18	High-pressure homogenization	/	Tween 80	/	/	(Pardeike & Müller, 2010)
25	Hesperidin	Wet media milling	/	poloxamer 188	Spray drying	PVP K25	(Wei et al., 2018)
26	Naproxen	Wet media milling	HPMC E15	Tween 80	Spray drying	Trehalose; lactose	(Kumar et al., 2015)
27	Carvedilol	Wet media milling	HPC-SL	SLS	Spray drying; Freeze drying	Mannitol	(Medarević et al., 2018)
28	Meloxicam	High-pressure homogenization; wet media milling; antisolvent precipitation	PVPk-17	/	Freeze drying	Mannitol	(Liu et al., 2020)
29	Meloxicam	Wet media milling	PVA	/	/	/	(Bartos et al., 2016)
30	Deacety mycoepoxydiene	High-pressure homogenization,	HPMC; PVP	Lecithin; poloxamer 188,	Freeze drying	Mannitol	(Wang et al., 2011)
31	Zileuton	Wet media milling	KollidonVA64 fine	Dowfax2A1	Spray drying	Mannitol; trehalose	(Jog & Burgess, 2019)
32	Aprepitant	h96 (lyophilization and high-pressure homogenization)	PVA	Tween 80; Poloxamer 188; SLS	/	/	(Kalvakuntla et al., 2016)
33	Aprepitant	Wet media milling	HPC-SSL; HPMC	SLS;	Spray drying; Freeze drying	Sucrose; mannitol	(Toziopoulou et al., 2017)
34	Baicalein	High-pressure homogenization	PVPK30; HPMC	PNS; Tween 80	Spray drying; Freeze drying	Sucrose; trehalose; lactose	(Xie et al., 2016)
35	Baicalein	High-pressure homogenization	/	Poloxamer 188	/	/	(Pi et al., 2019)
36	Atorvastatin	Antisolvent precipitation	/	Chitosan	/	/	(Kurakula et al., 2015)
37	Baicalin	High-pressure homogenization	HPMC	Poloxamer 188; TPGS	Freeze drying	Glucose; sucrose; lactose; trehalose; mannitol; sorbitol; PEG 4000	(Yue et al., 2014)
38	Valsartan	High-pressure homogenization	/	Poloxamer 188	Freeze drying	Mannitol	(Gora et al., 2016)
39	Flurbiprofen	High-pressure homogenization	HPMC; PVP K30	Tween 80; Plantacare 2000	Freeze drying	/	(Oktay et al., 2018, 2019, 2020)
40	Ritonavir	High-pressure homogenization	HPMC	SLS	Freeze drying	Mannitol	(Karakucuk et al., 2016)

Regardless of the solidification method, it is important to preserve the properties of the nanocrystal particles after the removal of water from the nanocrystal suspension. The influence of the redispersibility of nanocrystals after curing is a major concern. Dispersants (protectants) are typically added to nanosuspensions to maintain the redispersibility of the nanocrystals in water after solidification (Van Eerdenbrugh et al., 2008). Most protectants are water-soluble, such as mannitol, sucrose, lactose, and water-soluble polymers such as hydroxypropyl methyl cellulose (Dan et al., 2016; Parmentier et al., 2017). When the dry powder comes into contact with the water medium, the protective agent around the nanoparticles dissolves rapidly, thereby releasing the nanocrystals and maintaining them in their original dispersed state.

In a study on the preparation of fenofibrate nanocrystals, Zuo et al (2013) found that the average particle size of fenofibrate redispersion increased to 3901 nm without the addition of a protective agent, which was 6 times the particle size before drying. This means that irreversible aggregation occurs during the drying process, and, hence, the dry powder can no longer disperse into nanoparticles of the original size. A water-soluble dispersant can form a bridge that connects hydrophilic excipients to nanocrystals. When spray drying was conducted via the addition of protective agents (lactose, sucrose, glucose, maltose, and mannitol), the fenofibrate redispersibility was substantially improved, among which mannitol was the most effective protective agent for maintaining the redispersibility of the nanocrystals.

Teeranachaideekul et al. (2008) studied the particle sizes after freeze drying of nanosuspensions with and without cryoprotectants, and the results demonstrated that the average particle size of nanocrystals without cryoprotectants exceeded that of nanocrystals with cryoprotectants. In a study of naproxen nanocrystal spray drying, Kumar et al. (2015) found that lactose and trehalose could effectively inhibit the aggregation of nanoparticles. Ultimately, trehalose was used as a naproxen nanocrystal powder due to its higher yield than lactose.

### 4.2. Characterization and evaluation of solid nanocrystal preparations

#### 4.2.1. Surface morphology

**Figure 7.**
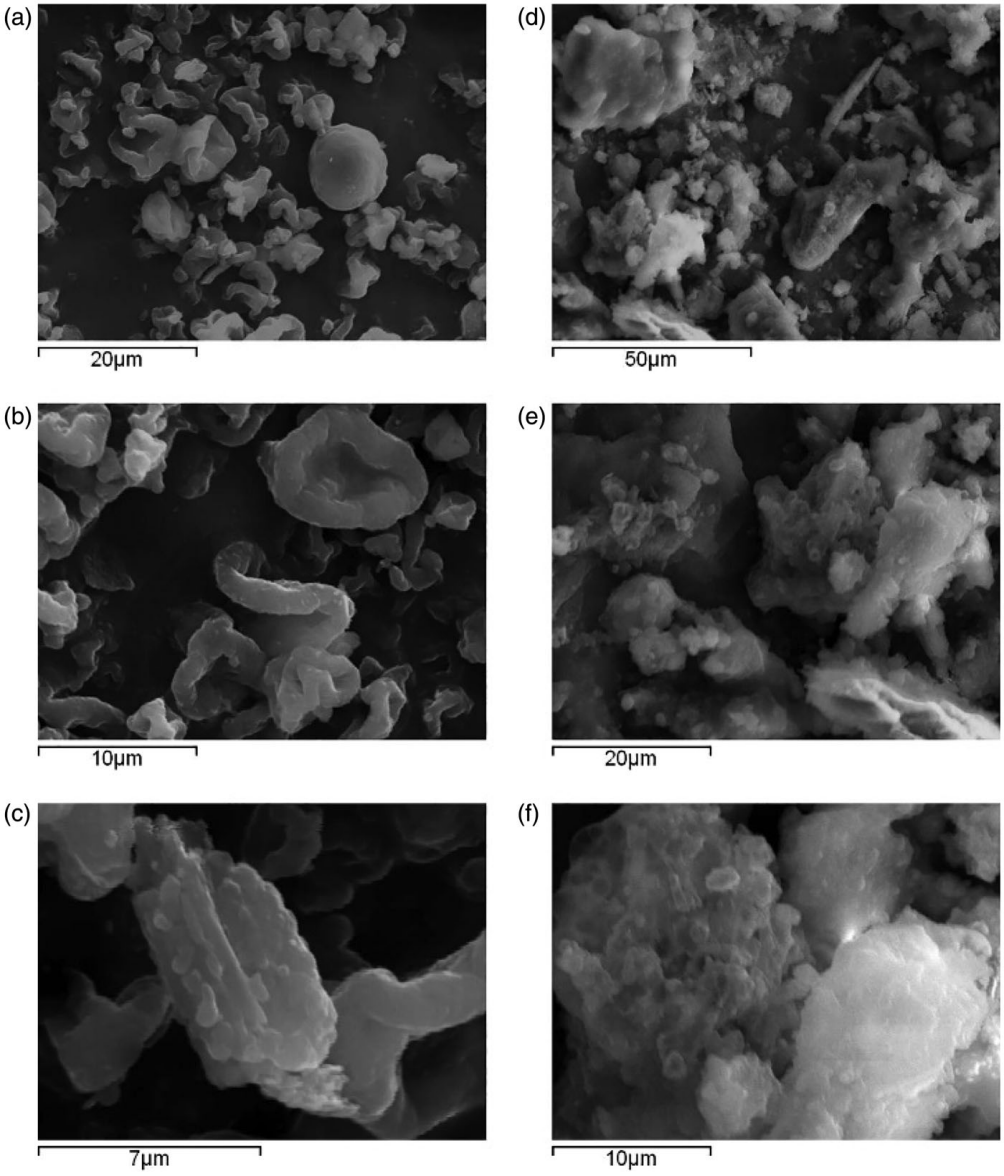
SEM photomicrographs of (a–c) a spray-dried CRV nanosuspension and (d–f) a freeze-dried nanosuspension. Reprinted with permission from Medarević et al. (2018). Copyright (2018) Elsevier B.V.

**Figure 8.**
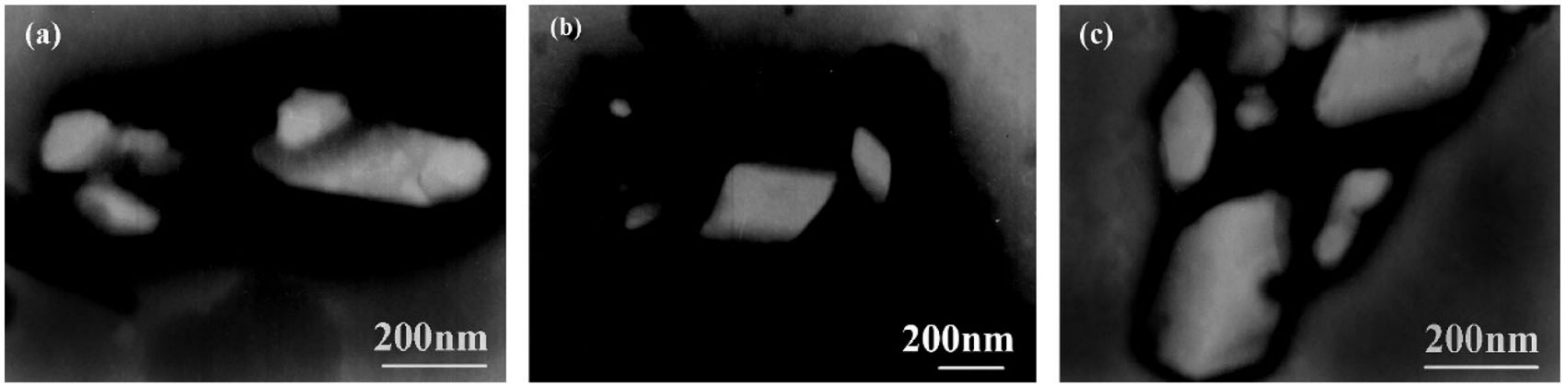
TEM images of (a) a fenofibrate nanocrystal suspension, (b) a redispersed suspension of a spray-dried powder in water and (c) a redispersed suspensions of tablets in water. Reprinted with permission from Zuo et al. (2013). Copyright (2013) Elsevier B.V.

The sizes and shapes of nanocrystals were analyzed via scanning electron microscopy (SEM) and transmission electron microscopy (TEM). In SEM, image results are generated through the interaction between the electron beam and atoms at various depths in the sample. For example, by collecting secondary electrons and backscattered electrons, information about the microstructure of the material can be obtained (Figure 7). In a transmission electron microscope, an image is obtained by capturing transmitted electrons in a sample. The accelerated and clustered electron beam can be transmitted to a very thin sample, and the electrons collide with the atoms in the sample and change direction, thereby generating solid angle scattering, which can be used to observe the ultrastructures of particles, and the resolution can reach 0.1 ∼ 0.2 nm (Figure 8).

#### 4.2.2. Crystal characteristics

The crystal characteristics of bulk drugs are highly attributes in the final products of nano pharmaceutical preparations. In the process of formation, the crystalline form of the drug may be changed due to external stresses and temperature changes. Although amorphous drugs have higher solubility, higher dissolution rates, or better compression properties, they are less physically and chemically stable than crystalline drugs, thereby resulting in uneven final product quality. Therefore, it is necessary to consider the crystal form changes before and after the formation of a drug. Nanocrystals can be characterized via differential scanning calorimetry (DSC), powder X-ray diffraction (P-XRD), Fourier-transform infrared spectroscopy (FTIR), and Raman spectroscopy.

DSC is a method of thermal analysis. A curve that is recorded by a differential scanning calorimeter is called a DSC curve. The rate of absorption or exothermic heat of the sample, namely, the heat flux rate (dH/dt), is selected as the ordinate, and the temperature (T) is selected as the abscissa. The endothermic peak, which can be readily observed in the DSC diagram, represents the energy consumption and is used to determine the melting point of the corresponding nanocrystal. The amorphous material shows no readily observable melting point peak but shows a glass transition temperature. Nanocrystals with smaller particle size are closer to the amorphous state and, therefore, have lower melting point peaks compared with the bulk drug crystals. P-XRD is another method for evaluating the crystal forms of nanocrystals. In some cases, the X-ray diffraction pattern of the nanocrystals may also show reduced or no peaks due to partial or complete amorphous formation of the nanocrystals during the grinding process (Zhang et al., 2007). Infrared spectroscopy is based on the differences in the infrared characteristic absorption spectra among functional groups in a material structure. When a reaction occurs between two components, the infrared absorption peak displacement or peak intensity change is generated, which is used to identify the molecular interaction between the two components. Raman spectroscopy is a type of molecular vibration spectroscopy that is based on inelastic light scattering. Its analysis principle is similar to that of infrared spectroscopy, but infrared signals are produced mainly by asymmetric vibration and polar groups. Therefore, by combining the results of Raman and infrared spectroscopy, the interaction between the drug and excipient in a nanocrystal preparation can be investigated at the molecular level, and a more comprehensive judgment can be obtained (Doyle, 1992).

Zuo et al. (2013) evaluated the crystal morphology of a sample with DSC and P-XRD. The DSC thermal image shows that the heat absorption peaks of the spray powder and tablet are shifted slightly forward, which may be because the drug is partially transformed into an amorphous form in the process of crushing or micro pulverization; the particle size reduction of the fenofibrate crystal may also cause the heat absorption peak to shift forward. With the crystallinity of fenofibrate bulk drug as 100%, the crystallinities of fenofibrate in the spray drying powder and tablet are approximately 95% and 73%, respectively. An X-ray diffraction (P-XRD) image showed that fenofibrate crystal I was retained in both the spray drying powder and the tablet but the crystalline transformation of mannitol occurred during spray drying, which was consistent with the DSC results that are presented above.

**Figure 9.**
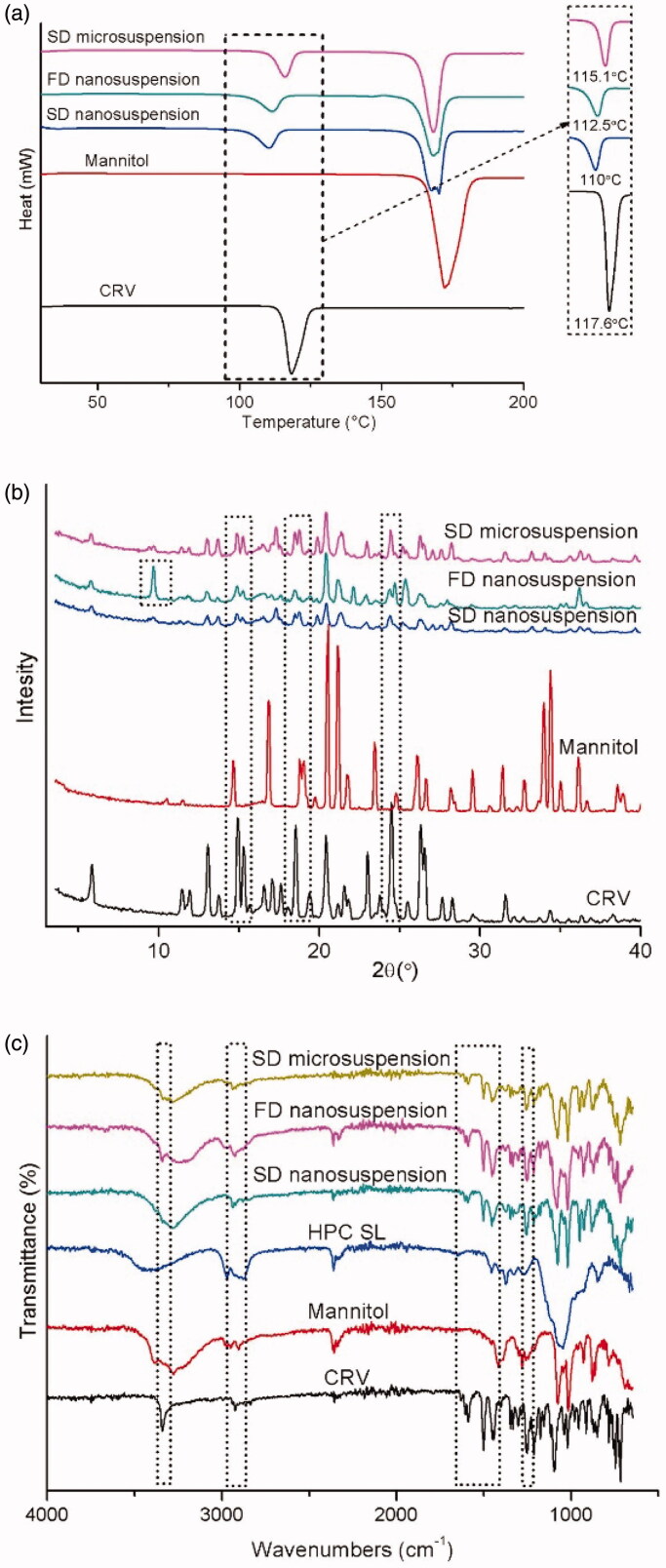
a. DSC thermograms of raw materials and prepared spray dried (SD) and freeze dried (FD) systems. b. PXRD patterns of raw materials and prepared SD and FD systems. c. FT-IR spectra of raw materials and prepared SD and FD systems (Medarević et al., 2018). Reprinted with permission from Medarević et al. (2018). Copyright (2018) Elsevier B.V.

According to a DSC thermal image that was obtained in a study that was conducted by Medarević et al. (2018), carvedilol showed a shift of the absorption peak and a decrease of the melting point after freeze drying or spray drying. Since thermal stress during the analysis will lead to a polymorphic transition, DSC technology cannot accurately identify the polymorphic transitions of materials. Therefore, according to P-XRD analysis results, neither wet grinding nor spray drying will cause polymorphic transitions of materials, while carvedilol will undergo crystal transformation during freeze drying. In combination with FTIR technology, the crystal type of carvedilol was identified, and there was no interaction between carvedilol and the functional groups of the stabilizers, such as HPC-SL and mannitol (Figure 9). In the process of nanocrystal drug development, multiple crystal characterization techniques can be combined to jointly investigate the possible crystal transformations and interactions in the preparation process of drug nanocrystals.

#### 4.2.3. *In vitro* and *in vivo* drug release studies

The drug release rates of drug nanocrystals are evaluated via an in vitro drug release study. The dissolution medium may be selected from among the pharmacopeia standard dissolution media or according to the solubilities of the drug in various media. The particle size of the nanocrystals determines the overall dissolution rate. Since nanocrystals have higher dissolution rates and larger ratios of surface area to volume, smaller particles have higher dissolution rates than larger particles. The dissolution rates of nanocrystals can also be controlled by applying a coating of hydrophobic polymers.

Due to the diversity and heterogeneity of nanocrystal preparations and the complexity of in vivo release behavior, the establishment of an effective in vitro dissolution method for predicting in vivo release behavior remains a technical challenge. Kumar et al. (2014, 2015) used the dialysis sac method, which was developed in the previous stage, to conduct an in vitro release test. Samples were obtained at a predetermined time interval, and HPLC quantitative analysis was conducted to draw the dissolution curve. This method can distinguish among sizes of nanocrystals and obtain the release curves for various sizes. Sievens-Figueroa et al. (2012) prepared a griseofulvin nanosuspension and compared the performances of the basket method and the flow-through cell method in vitro drug release. The results demonstrated that the flow-through cell method outperformed the basket method. He et al. (2015) prepared teniposide nanosuspensions for intravenous administration. They used the dialysis bag method to compare the in vitro release of teniposide nanosuspensions freeze-dried preparation and the marketed preparation. The results revealed that the passage of teniposide molecule in the nanosuspensions through the dialysis membrane was considerably slower as compared with that of marketed preparation. The slow release rate of teniposide nanosuspensions could be attributed to the slowly solution of teniposide, which maybe add to the benefit of prolonging the system circulation of teniposide for chemotherapy.

In vitro release tests are crucial in preparation development and quality control. In addition to dialysis and the flow-through cell method, there are sampling and separation, gel, pressure ultrafiltration, turbidimetric analysis, and in situ methods (Crisp et al., 2007; Dai et al., 2007; Xia et al., 2010; Anhalt et al., 2012; Kumar et al., 2014; Xie et al., 2016; Liu et al., 2019). The researchers proposed that the in vitro release method for nanodrug delivery systems could be improved by introducing in vivo proteins into the in vitro release medium to design and simulate the distribution characteristics of the drug delivery system in vivo (Liu et al., 2019). Many methods have been reported, and each has advantages and disadvantages. In the process of nano-formulation development, suitable dissolution equipment should be selected according to the drug properties, dosage forms, and formulation process. Reasonable dissolution medium conditions should also be identified to develop suitable dissolution methods in vitro (Nothnagel & Wacker, 2018). The proposed dissolution method, which has distinguishing power, can screen for the desired formulation, optimize the technological parameters during the research process, and provide a reasonable reference for prescription evaluation.

The optimal formulation is selected through in vitro dissolution to optimize the formulation and process parameters. Then, the drug release is studied in vivo to evaluate the bioavailability of the drug. Many research groups have studied the in vivo properties of nanocrystals by administering them to rats or mice through various routes. Guo et al. (2015) studied the in vivo performance of the rebamipide nanocrystal. They observed that the C_max_ and AUC_0–24 h_ values of rebamipide nanocrystals were 1 and 1.57 times larger than those of the marketed preparations; hence, the nanocrystals significantly improved the bioavailability of the drug.

However, if an effective in vitro and in vivo correlation (IVIVC) can be established, the number of experiments in vivo will be reduced significantly. IVIVC is a mathematical relationship between in vitro feature of the product (for example dissolution rate) and in vivo performance (Rettig & Mysicka, 2008). The major objective of IVIVC is to be able to use in vitro data to predict in vivo performance serving as a surrogate for an in vivo bioavailability test and to support biowaivers (Gonzalez-Garcia et al., 2015). Karakucuk et al. (2019) prepared ritonavir nanosuspension with microfluidization method. In vitro dissolution and in vivo bioavailability of nanosuspension were evaluated in the research. In nanosuspension formulation, the dissolution and solubility were improved which caused higher correlation between in vitro dissolution and in vivo pharmacokinetic data. Ghosh et al. (2012) conducted in vivo pharmacokinetic experiments with beagle dogs and found that there was a significant correlation between the particle size and bioavailability of drug molecules. As the dissolution rate increased, AUC and C _max_ increased significantly when the drug was converted to nanocrystals. Nanosuspension with narrow distributions of particles produced systems with improved absorption, less variability, and superior stability by minimizing the Ostwald ripening process. Imono et al. (2020) prepared microsuspensions of two model drugs, namely, fenofibrate and megesterone acetate, along with three nanosuspensions with various particle sizes. Through in vitro dissolution-permeation studies and in vivo oral pharmacokinetic studies, it was found that the particle size reduction only slightly increased the apparent solubilities (1.4 times) but significantly increased the penetration rates of the two drugs (3 times). A strong positive correlation was identified between the in vitro permeation rate and the in vivo maximum absorption rate. The permeability increase due to the formation of nanocrystals is the main factor for improving the oral absorption, and the dissolution permeability in vitro can be used to predict the oral absorption enhancement of nanocrystals.

The absorption mechanism of parenteral nanocrystal drug delivery is complex and diverse, which also brings great challenges to the study of nanocrystal drug release in vitro (Alexis et al., 2008). For example, intravenously administered nanocrystal formulations are a new type of therapeutics, which encounter a rather complex and dynamic in vivo environment. As a consequence, it is difficult to establish the IVIVC for these formulations and only few success stories have been published so far. Jablonka et al. (2019) established an IVIVC for the drug formulation Foscan^®^ on the basis of in vitro release and particle characterization data. Furthermore, the extrapolations made by the physiologically based pharmakokinetic and biodistribution model generates an expected in vivo biodistribution pattern based on early preclinical in vitro and in vivo data. In brief, establishing in vitro–in vivo correlation of nanocrystals can be used to well predict the in vivo behavior of drugs, elucidate the absorption mechanism and reduce the risk of clinical drug use (Bao et al., 2017; Litou et al., 2019).

## 5. Conclusions

Particle size instability has always been a major technical limitation in the development of nanocrystal drugs. The problems that are associated with nanocrystal drug instability include aggregation, Ostwald ripening, and sedimentation. The stability depends on the interactions between drug nanocrystals and the surface free energy, among other factors. The interactions between drug nanocrystals and stabilizers have yet to be fully understood, and the results cannot be clearly explained by established knowledge. The reason may be that the stability of drug nanocrystals is influenced by various factors, such as the physical and chemical properties of the nanocrystals, stabilizers, dispersion media, and surrounding environment, including temperature. Therefore, it is necessary to identify the most suitable stabilizer and prescription variables experimentally according to various action mechanisms and influencing factors. In addition, nanocrystal preparations still face major technical challenges, especially in the control of the effects of solidification on the physical stability and redispersibility. In vitro and in vivo evaluation and other aspects still need to be continuously explored to develop scientific and standardized preparation and evaluation methods.
